# Climate change and the rising threat of vector-borne diseases in the Andes

**DOI:** 10.1016/j.onehlt.2026.101362

**Published:** 2026-02-13

**Authors:** Esteban Ortiz-Prado, Jorge Vasconez-Gonzalez, Jean Carlo Pazmiño-Almeida, Mathias Rafael Serrano-Núñez, Esteban Acosta-Muñoz, Johana Sofía Sánchez-Bustamante, Camila Salazar-Santoliva, Ana Paula Bastidas, John Alexander Altamirano-Castillo, Sofia Vanessa Villacis-Pauta, Juan S. Izquierdo-Condoy

**Affiliations:** One Health Research Group, Universidad de Las Américas, Quito, Ecuador

**Keywords:** Altitudinal spread, Shifting distribution, Andean highlands, Anthropogenic drivers, Climate-sensitive surveillance

## Abstract

Vector-borne diseases such as dengue, malaria, leishmaniasis, and Chagas disease continue to cause millions of infections and thousands of deaths each year, particularly in low- and middle-income regions of South America. In recent years, climate change has profoundly altered the distribution and behavior of arthropod vectors, promoting their expansion into new ecological niches, including high-altitude areas of the Andes once considered unsuitable for transmission. Countries such as Colombia, Ecuador, Peru, and Bolivia have reported outbreaks of dengue and malaria in populations residing above 2000 m above sea level, revealing an unprecedented epidemiological shift. Importantly, this emerging scenario reflects more than an environmental process: it also exposes deep social vulnerabilities linked to poverty, deforestation, and limited access to health services, which can magnify outbreak impacts and constrain timely response. Within this context, the climate–vector–inequity triad offers an integrative perspective to understand how climatic and structural factors converge to amplify risk. Addressing this challenge requires altitude-sensitive surveillance systems, ecosystem restoration, and health policies grounded in the One Health approach to strengthen prevention, diagnosis, and response capacity in highland regions of the Andes, particularly in vulnerable indigenous and rural communities across Ecuador, Colombia, Peru, and Bolivia where socioeconomic disparities exacerbate transmission risks.

## Highlights

• Vectors have expanded to higher-altitude areas due to the influence of climate change.
• Andean countries have reported cases of vector-borne diseases in regions where they previously did not exist.
• It is necessary to establish entomological surveillance in high-altitude areas.

## Graphical abstract

**Figure:**
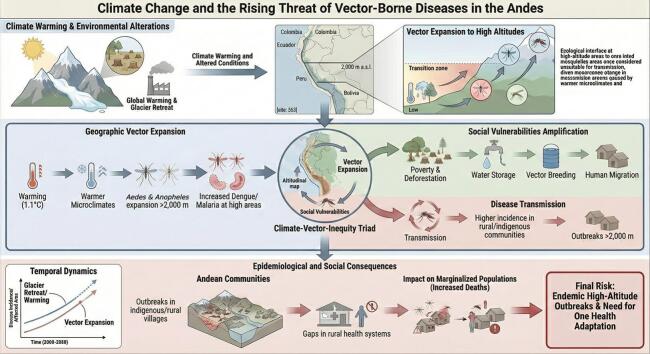

## 1 Introduction

Vector-borne diseases are infections caused by viruses, parasites, or bacteria that are transmitted by invertebrate vectors, most commonly hematophagous arthropods such as mosquitoes, ticks, fleas, or sandflies—that act as intermediate hosts and carry pathogens between vertebrate hosts [1,2]. Depending on the pathogen–vector–host system, transmission may occur between humans, between animals, or between animals and humans [3]. Currently, these diseases cause more than one billion infections annually and approximately one million deaths. The greatest burden falls on populations living in low-income countries, where resources for disease control are limited [4,5].

The planet's diverse climatic zones have distinctly shaped the distribution of vectors and associated diseases, particularly in tropical regions. Humid climates, rainforests, and increasingly encroaching agricultural frontiers bring humans into closer contact with reservoirs, water sources, and wild animals, such as monkeys that transmit yellow fever [6,7]. However, as global progress continues driven by late industrialization, globalization, population growth, and greenhouse gas emissions the planet is warming at an alarming rate, consistent with historical records [8]. According to the Intergovernmental Panel on Climate Change (IPCC), human-induced global warming has reached approximately 1.1 °C above pre-industrial levels, with the rate of warming since 1982 accelerating to 0.20 °C per decade [9]. This warming has led to significant glacier retreat, with global glaciers losing an estimated 6.54 trillion tons of ice from 2000 to 2023, contributing 18 mm to sea level rise, at an annual rate of 0.75 mm [10]. In the Andes, this has been particularly acute, with tropical glaciers in Ecuador and Peru retreating by 30–50% since the 1980s, creating warmer microclimates and expanding vector habitats in regions like the Pichincha highlands and Cusco valleys [11]. The rate of glacier mass loss has accelerated by 36% from 2012 to 2023 compared to 2000–2011, with 2023 alone recording a loss of 80 billion metric tons, equivalent to 6% of the total loss since 1975 [10,12]. This rapid glacier melt has expanded the habitats of mosquitoes and other vectors into areas previously unsuitable, such as higher altitudes. Climate change is a significant risk factor that profoundly influences the transmission dynamics, geographic distribution, and resurgence of vector-borne diseases [13].

This shift is consistent with regional warming and hydroclimatic changes, including increases in temperature across latitudinal and altitudinal gradients and changes in precipitation patterns, which can relax climatic constraints on vector survival and development [14]. In the Andean region, *Aedes aegypti* has been documented at elevations previously considered low risk, including high-altitude settings where cooler ambient temperatures historically limited mosquito development and survival [15,16].

Climate-induced altitudinal shifts of vector-borne diseases in the Andes have been increasingly documented [17,18]. We propose that these shifts represent not merely an ecological redistribution of arthropod vectors, but a social vulnerability amplifier that disproportionately affects marginalized highland populations. This phenomenon—driven by the interplay between climate warming, ecosystem degradation, and structural inequities—demands a new paradigm of altitude-sensitive health system adaptation.

This manuscript introduces the concept of the ‘climate–vector–inequity triad,’ a transdisciplinary framework linking climatic gradients, ecological plasticity, and social determinants of health, which can become an essential tool for guiding epidemiological surveillance priorities and informing health policy decision-making. By synthesizing evidence across ecological, epidemiological, and socioeconomic dimensions, we argue that the Andean highlands are increasingly recognized as a sentinel biome for the study of climate-sensitive vector-borne disease dynamics and resilience strategies.

## 2 The expanding reach of vectors

Evidence shows that climate warming alters the global distributions of mosquitoes that transmit pathogens; rising temperatures have enabled the geographic expansion of vectors such as *Aedes aegypti* and *Aedes albopictus*, allowing disease transmission at higher latitudes [14,19]. Meanwhile, changes in rainfall patterns, humidity, and extreme weather events also play a critical role in influencing mosquito breeding sites and viral replication rates [[20], [21], [22]]. On the other hand, deforestation, regardless of its cause, leads to habitat fragmentation and loss, reducing the availability of essential resources such as food and water. As a result, wildlife is forced to move into urbanized areas, increasing contact with domestic animals and humans and facilitating the transmission of pathogens between wildlife and human populations [23].

In Colombia, *Aedes aegypti* was historically not reported above 1585 m a.s.l., yet its presence has now been documented at 2302 m a.s.l. (Table 1) [16]. In Ecuador, using national larval surveillance data and ecological niche modeling, high-elevation Andean areas appear largely unsuitable under current conditions; however, 2050 projections across emissions scenarios suggest a shift in suitability toward Andean transitional elevations, with up to 4215 km^2^ affected and > 12,000 residents potentially exposed under the most extreme scenario [17]. In 2024, South America experienced a dengue crisis that coincided with heavy rainfall, widespread flooding, and the 2023–2024 El Niño period [24]; recent multi-country quantitative analyses using teleconnection metrics and distributed-lag models support a significant association between ENSO fluctuations and dengue dynamics, including estimates of excess cases during the 2023–2024 event [25]. With respect to malaria, climate modeling studies indicate that rising temperatures may extend transmission windows in highland areas by approximately 1.6 months. Importantly, these modeling estimates require verification using field surveillance data [26]. Furthermore, in Bolivia, the presence of *Anopheles pseudopunctipennis*, a malaria vector, and cases of the disease were reported in high-altitude populations (2615–3592 m a.s.l.), regions where malaria had not previously been recorded. This event was associated with an increase in average atmospheric temperature of 0.85 °C [27,28].

**Table 1:** Empirical observations and model-based projections related to altitudinal expansion of vectors and vector-borne diseases in the Andes.

Author	Year	Country/region	Vector/species studied	Results	Evidence type
Ruiz-Lopez et al. [16].	2016	Colombia	*Aedes aegypti*	*Aedes aegypti* recorded up to 2302 m a.s.l.; natural DENV-2 infection detected at 1984 m a.s.l. (RT-PCR)	Field entomological evidence (ovitrapping/immature-to-adult emergence) and Field virological evidence (RT-PCR)
Lippi et al. [17].	2019	Ecuador	*Aedes aegypti*	ENM using MSP larval presence data projects a 2050 shift in habitat suitability toward Andean transitional elevations, with up to 4215 km^2^ becoming suitable under the most extreme scenario.	Model-based evidence (ecological niche modeling) using routine larval surveillance data.
Rutar et al. [27]	2004	Bolivia	*Plasmodium vivax*	First documented malaria outbreak in a highland community at 2300 m a.s.l.; 52/63 blood smears positive for *P. vivax*	Outbreak investigation / field epidemiology (descriptive observational study) with laboratory confirmation
Pinault & Hunter [18]	2011	Ecuador	*Anopheles albimanus, Anopheles pseudopunctipennis, Anopheles punctimacula, Anopheles eiseni* and *Anopheles oswaldoi s.l*	*Anopheles punctimacula* and *Anopheles oswaldoi s.l*. are encroaching into higher altitude regions, in some cases reaching higher maximum altitudes (1541 m, 1906 m, and 1230 m, respectively).	Field entomology / vector surveillance study
Echeverria-Cárdenas et al. [29]	2021	Colombia	*Aedes albopictus*	The vector is estimated to occur across 96% of continental Colombia up to 3000 m a.s.l. (∼48 million people at risk). Under the RCP 2.6 scenario, its distribution could cover ∼90% up to 3100 m a.s.l. by 2050 and 2070.	Predictive modeling study: ecological niche modeling (MaxEnt) using occurrence records (native range + South America) and bioclimatic variables.
Requena-Zuñiga et al. [30]	2016	Peru	*Aedes aegypti*	Field larval inspections and collections confirmed *Aedes aegypti* in Ica and Huánuco; the report highlights detection at ∼1900 m a.s.l. in Huánuco	Entomological field detection / surveillance report
Gomez et al. [31]	2014	Ecuador	*Lutzomyia ayacuchensis*	The distribution of *Lu. ayacuchensis* was identified at higher altitude and the ratio is increased with increasing altitude.	Entomological field survey across an altitudinal gradient (descriptive observational study) with parasite detection in vectors (infection screening)
Castillo-Quino et al. [32]	2018	Boliva	*Aedes aegypti*	Colonization of *Aedes aegypti* at altitudes above 2200 m a.s.l.	Public health entomological surveillance report

Recent studies highlight the remarkable physiological plasticity of *Aedes aegypti* and *Anopheles* spp., enabling their survival and reproduction in cooler, high-altitude environments once deemed unsuitable [33,34]. This adaptability is mediated by temperature-dependent gene expression changes, including upregulation of heat shock proteins (HSP70, HSP90), modulation of cuticular hydrocarbon composition, and alterations in metabolic rate that reduce developmental thresholds [35]. Experimental evidence indicates that *Aedes aegypti* populations can now complete their life cycle at mean daily temperatures of 15–17 °C, previously considered non-viable, demonstrating rapid evolutionary plasticity under sustained warming pressures [22]. These findings suggest that the vector's altitudinal expansion is not merely a passive response to climate, but an active evolutionary adaptation, underscoring the need for integrative entomological monitoring at high elevations.

Additionally, livestock production has been described as a factor that may increase mosquito populations, and pigs, horses, dogs, and cats can serve as sentinel animals for arboviruses [36]. In Andean contexts, such as smallholder farms in rural Ecuador and Bolivia, where mixed livestock-human habitation is common due to limited land availability, this proximity may amplify zoonotic spillover risks, as evidenced by serological studies in highland communities showing arbovirus antibodies in domestic animals [37,38]. Presence of dengue virus has been detected in birds, horses, bovines, and pigs [39]; however, studies assessing the influence of livestock farming or poultry rearing on the expansion of arboviruses in the Andean region have not been identified. Studies conducted in other countries have not found a significant association between livestock rearing and the presence of *Aedes* spp. mosquitoes, nor between livestock rearing and dengue incidence [40].

## 3 The problem of the Andes

Deforestation is responsible for disrupting ecosystem dynamics and generating new breeding habitats for disease vectors [18,41]. An analysis of 87 mosquito species from 12 countries revealed that approximately 52.9% were associated with deforested habitats; moreover, among the species favored by deforestation, 56.5% were confirmed vectors of human pathogens, including *Anopheles*, *Aedes*, and *Culex* [41]. Deforestation can alter habitats, creating favorable microenvironments that support the growth of vector populations; it has been reported that pools in deforested areas exhibit lower salinity and acidity, making them more suitable for the larval development of certain *Anopheles* species [42]. Vittor et al. reported in their study in Peru that deforested areas exhibited a biting rate of *Anopheles darlingi* more than 278 times higher than that recorded in predominantly forested areas [43]. Moreover, ecological disturbances influence the emergence and proliferation of zoonotic parasitic diseases such as leishmaniasis, as the replacement of forests with crops, livestock, and small animal farming can create favorable habitats for parasites and their vector hosts [42].

Low educational attainment, minority status or residence in underserved communities, and the absence or inadequacy of public services further act as barriers to disease prevention [44]. The spread of leishmaniasis in the Peruvian highlands, for instance, has placed additional strain on under-resourced health systems, underscoring the intersection of climate change and social inequity [45,46]. Highland communities often indigenous, rural, and peri-urban—face structural barriers: intermittent piped water (promoting storage), limited entomological capacity, and geographic isolation [[47], [48], [49], [50], [51], [52]]. In regions like the Ayacucho valley in Peru or Loja province in Ecuador, where indigenous groups such as the Quechua and Saraguro rely on seasonal migration between altitudes, these barriers delay detection of sporadic cases, potentially leading to localized outbreaks rather than sustained transmission, as seen in recent Pan American Health Organization reports [53]. Introducing vector-borne diseases into health systems designed for different burdens risks diagnostic delay, higher case severity, and late outbreak detection. Climate adaptation in health must therefore prioritize service continuity (water, waste), early-warning integration, and clinical preparedness at altitude.

## 4 Toward mitigation and adaptation

Effective responses require integrated, adaptive strategies. Strengthening surveillance in high-altitude regions is critical for early detection of vector shifts and outbreak preparedness. Community-based vector control measures, such as the elimination of standing water and deployment of insecticide-treated nets, remain essential tools [54,55]. On the other hand, the increase in insecticide resistance is becoming one of the main challenges for controlling vector-borne diseases, making it necessary to have robust surveillance systems, sustained investment in innovative tools, and integrated vector management approaches that combine chemical, biological, and genetic methods. [56,57].

It is also important to note that geospatial technologies provide tools to monitor environmental changes and predict disease transmission patterns. However, it is necessary to improve data accessibility, foster cross-sector collaboration, and include socioeconomic variables [58]. At the same time, ecosystem restoration through reforestation and sustainable land-use practices can help re-establish ecological balances and limit vector habitats [59,60]. Crucially, public health policies in Andean countries must embed climate adaptation measures, ensuring that healthcare systems and professionals are prepared to address vector-borne diseases in previously unaffected regions.

## 5 Policy and practice implications

Policy and practice responses must account for the unique ecosystems of the Andes and the accelerating upslope migration of vectors. Developing altitude-sensitive early warning systems is critical. These systems should integrate microclimatic indicators such as minimum temperature, dew point, and precipitation anomalies linked to ENSO events with urban service data like water supply continuity. Such coupling would enable forecasting of upslope suitability weeks in advance, providing valuable lead time for interventions.

Equally important is the implementation of targeted source-reduction strategies in peri-urban belts located between 2000 and 3200 m above sea level. Measures such as container management, ovitrap networks, and rapid larval source elimination prior to the warm and rainy seasons can substantially reduce outbreak risk.

At the same time, highland health systems must strengthen their surge clinical capacity. Pre-positioning diagnostic tools and standardized case management protocols for dengue and malaria in facilities that have historically seen low caseloads can help prevent delayed diagnoses and overwhelmed services. Land-use planning also presents opportunities for co-benefits: reforestation and green infrastructure not only mitigate urban heat islands and runoff but also help reduce vector habitats while aligning with biodiversity goals **[****61****]**. Finally, mobility-informed control strategies are essential. Mapping daily and seasonal flows between lowlands and highlands can anticipate seeding events and guide targeted interventions in vulnerable communities. For instance, in the Andean corridor spanning Colombia, Ecuador and Peru, where informal trade and migration routes facilitate vector movement, cross-border initiatives like the Andean Community's health protocols could integrate ENSO forecasting with community-led surveillance **[****62****,****63****]**.

## 6 Limitations

This work is a narrative review and does not present new primary analyses. No original datasets were generated or statistically reanalyzed; therefore, the relationships discussed between climate variability (e.g., warming trends, precipitation anomalies, ENSO) and vector-borne disease patterns in the Andes are based on previously published studies and public reports and should not be interpreted as causal estimates from this manuscript. In addition, surveillance capacity and diagnostic confirmation in highland and remote settings remain uneven, so both vector presence and disease occurrence may be under detected or inconsistently reported and therefore, data is not available. Finally, several interacting drivers such as human mobility, land-use change at fine spatial scale, local water-service intermittency, and evolving insecticide resistance cannot be quantified uniformly within the existing literature and may confound or modify the apparent climate disease signal.

## 7 Conclusions

The upward expansion of vector-borne diseases into the Andean highlands is not only an ecological consequence of climate change but also a reflection of deep social and environmental inequities that amplify vulnerability across mountain communities. Rising temperatures altered precipitation patterns, and deforestation have expanded the range of vectors such as *Aedes aegypti* and *Anopheles* species, allowing diseases like dengue, malaria, and leishmaniasis to emerge in previously unaffected high-altitude regions. These environmental changes, compounded by weak health infrastructure and socioeconomic disparities, have created new zones of vulnerability for indigenous and rural populations.

Addressing this challenge requires a transdisciplinary One Health strategy that integrates climate science, ecology, and public health. Priorities include strengthening high-altitude surveillance through the integration of environmental, veterinary, and human health monitoring, restoring ecosystems via reforestation and sustainable land use, and enhancing diagnostic and clinical capacity within local health systems. The implementation of altitude-sensitive early warning systems, which incorporate microclimate and urban service data, can improve outbreak preparedness, while community-based control measures, such as container management and ovitrap networks, remain essential for prevention. This integrated approach ensures that shared risks across humans, animals, and the environment are detected early and managed effectively, taking into account the unique challenges of high-altitude ecosystems.

Building resilience against this emerging threat will depend on embedding climate adaptation into national health policies, ensuring equitable access to care, and promoting sustained investment in Andean public health research. Swift, coordinated, and equity-centered action is essential to protect highland populations and prevent these diseases from becoming permanently established.

## CRediT authorship contribution statement

**Esteban Ortiz-Prado:** Writing – review & editing, Validation, Supervision, Project administration, Methodology, Investigation, Funding acquisition, Conceptualization. **Jorge Vasconez-Gonzalez:** Writing – review & editing, Visualization, Validation, Supervision, Project administration, Methodology, Investigation, Funding acquisition, Formal analysis, Data curation, Conceptualization. **Jean Carlo Pazmiño-Almeida:** Writing – original draft, Resources, Methodology, Investigation, Formal analysis, Data curation. **Mathias Rafael Serrano-Núñez:** Writing – original draft, Resources, Methodology, Investigation, Formal analysis, Data curation. **Esteban Acosta-Muñoz:** Writing – original draft, Visualization, Resources, Methodology, Investigation, Formal analysis, Data curation. **Johana Sofía Sánchez-Bustamante:** Writing – original draft, Visualization, Resources, Methodology, Investigation, Formal analysis, Data curation. **Camila Salazar-Santoliva:** Writing – original draft, Visualization, Resources, Methodology, Investigation, Formal analysis, Data curation. **Ana Paula Bastidas:** Writing – original draft, Visualization, Resources, Investigation, Formal analysis, Data curation. **John Alexander Altamirano-Castillo:** Writing – original draft, Visualization, Resources, Methodology, Investigation, Data curation. **Sofia Vanessa Villacis-Pauta:** Writing – original draft, Visualization, Software, Resources, Investigation, Data curation. **Juan S. Izquierdo-Condoy:** Writing – review & editing, Visualization, Validation, Supervision, Project administration, Methodology, Funding acquisition.

## Ethics statement

This study did not involve the collection of primary data or human participants. It is based entirely on the review and analysis of secondary, publicly available information. Ethical approval was therefore not required.

## Declaration of generative AI and AI-assisted technologies in the writing process

During the preparation of this work the authors used ChatGPT version 5.1 to improve readability and language. After using this tool/service, the authors reviewed and edited the content as needed and take full responsibility for the content of the published article.

## Funding

This study received funding from Universidad de Las Américas (Project MED.EOP.23.01).

## Declaration of competing interest

The authors declare that the research was conducted in the absence of any commercial or financial relationships that could be construed as a potential conflict of interest.

## Data Availability

No new datasets were generated or analyzed for this study. All data supporting the conclusions of this article are available in the cited references and publicly accessible reports from the World Health Organization, national health authorities, and previously published peer-reviewed studies.
